# Th@C_84_ Revisited: Lost in the Corners of One’s Own Cage

**DOI:** 10.1021/acs.jpca.6c00909

**Published:** 2026-05-04

**Authors:** Jakub Kaminský, Adam Jaroš, Michal Straka

**Affiliations:** † 89220 Institute of Organic Chemistry and Biochemistry of the Czech Academy of Sciences, Flemingovo nám. 2, Prague CZ−16610, Czech Republic; ‡ Department of Chemistry of Natural Compounds, University of Chemistry and Technology Prague, Technická 5, Prague 166 28, Czech Republic

## Abstract

The energetic ordering of actinide endohedral fullerene isomers depends sensitively on proper exploration of their intramolecular potential energy surfaces. In a previous theoretical study, we predicted that the Th@C_s_(10)–C_84_ isomer is the lowest-energy structure, which is in apparent contradiction with recent experiments identifying Th@C_2_(8)–C_84_ and Th@C_s_(15)–C_84_ as the preferentially formed isomers. Here, we theoretically reevaluate all 24 isolated-pentagon-rule Th@C_84_ isomers using systematic sampling of the thorium positions inside the carbon cage. We show that the experimentally observed isomers are indeed the lowest-energy structures when the conformational space is properly explored. The earlier misassignment is traced to convergence into higher-energy local minima caused by strong, directional Th–cage covalent interactions. Bonding analysis confirms the formal Th­(IV) oxidation state, and the revised UV–vis–NIR spectra are consistent with the experimental results. Our results emphasize the importance of extensive conformational sampling for the reliable theoretical characterization of actinide endohedral fullerenes.

## Graphical abstract

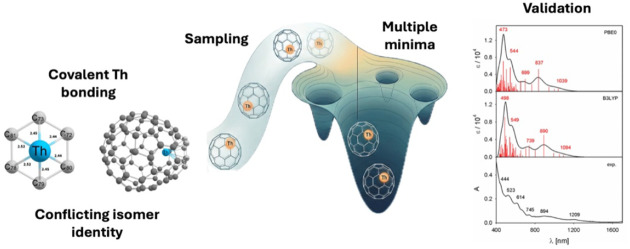

## 1 Introduction

The first thorium endohedral fullerene, Th@C_84_, was reported by Akiyama et al. in 2002 and identified by UV–vis–NIR and XANES spectroscopy as a Th^4+^@C_84_^4 –^ system.^1^ At the time, the cage structure could not be determined experimentally. Motivated by this uncertainty, we previously investigated a range of isolated-pentagon-rule (IPR) and non-IPR Th@C_84_ isomers using density functional theory and identified Th@*C*_*s*_(10)–C_84_ (a thorium atom enclosed in a in the *C*_*s*_-symmetric C_84_ isomer 10) as the lowest-energy structure.^2^

Recently, however, arc-discharge experiments combined with single-crystal X-ray diffraction unambiguously identified two different Th@C_84_ isomers, Th@*C*_2_(8)–C_84_ and Th@*C*_*s*_(15)–C_84_, as preferentially formed structures.^3^ This finding contradicts our previous theoretical prediction and raises a fundamental question concerning the reliability of the theoretical energetic ordering in actinide endohedral fullerenes.

Since the initial report of Th@C_84_, a broad family of thorium endohedral fullerenes has been synthesized and structurally characterized. These include Th@*C*_3_ *_v_* (8)–C_82_,^4^ Th@*C*_1_(28324)–C_80_, Th@*T*_*d*_(19151)–C_76_, Th@*C*_1_(11)–C_86_, and several Th@C_82_ and Th@C_86_ isomers, as well as non-IPR cages, such as Th@*C*_1_(17418)–C_76_.^4–11^ In all experimentally characterized mononuclear thorium fullerenes, spectroscopic, electrochemical, and theoretical analyses consistently indicate a formal Th­(IV) oxidation state accompanied by significant metal–cage covalency.

In addition to mononuclear species, binuclear and cluster fullerenes, such as Th_2_@*I*_*h*_(7)–C_80_ and ThC_2_@C_82_, have been reported,^12,13^ further highlighting the rich bonding behavior of thorium inside carbon cages. In these systems, strong actinide–cage interactions, back-donation effects, and direct actinide–actinide bonding have been demonstrated. These findings collectively show that thorium in endohedral fullerenes does not behave as a purely electrostatic guest but instead forms strong directional covalent interactions with the carbon cage.

In parallel with experimental advances, extensive theoretical studies have been devoted to thorium endohedral fullerenes. Density functional studies have explored Th@C_2*n*_ systems over a broad cage size range (2*n* = 64–88) and specific cage topologies, such as Th@*D*_3*h*_(14246)–C_74_ and Th@C_76_,^5,14–17^ revealing the pronounced off-center positioning of thorium and strong metal–cage covalent interactions. Computational studies have also predicted covalent Th–Th bonds in binuclear systems Th_2_@C_*n*_ (*n* = 60, 70, 80, 90),^18–20^ which was later corroborated experimentally for Th_2_@*I*_*h*_(7)–C_80_. In addition, alternative isomers of Th@C_78_, including cages containing heptagonal rings,^14^ have been proposed theoretically. More generally, the chemical bonding patterns in thorium and uranium endohedral fullerenes have been comprehensively reviewed, highlighting the central role of actinide–cage covalency in determining structure and stability.^21^

Such directional Th–cage bonding has important consequences for the intramolecular potential energy surface. The metal atom tends to localize near specific cage motifs, giving rise to multiple conformational minima for a single-cage isomer. These minima may be separated by substantial energy barriers, and geometry optimizations initiated from different metal positions atom may therefore converge to different local minima. Consequently, incomplete exploration of conformational space can lead to incorrect energetic ordering of isomers, even when otherwise reliable electronic-structure methods are employed.

In this study, we revisit the energetic ordering of all 24 IPR Th@C_84_ isomers, with particular emphasis on systematic sampling of thorium position inside the carbon cage. We demonstrate that the previously reported discrepancy with experiment originates from the convergence to higher-energy conformers rather than from an intrinsic failure of the underlying electronic structure methods. We further analyze the chemical bonding in the experimentally observed Th@*C*_2_(8)–C_84_ and Th@*C*_s_(15)–C_84_ isomers and reevaluate their UV–vis–NIR and ^13^C NMR properties, obtaining results consistent with the available experimental data.

## 2 Methods

### 2.1 Geometry Optimization

All optimizations were performed using Gaussian 16, Rev. C.01.^22^ For each of the 24 isolated pentagon rule (IPR) C_84_ cages, multiple starting structures were generated by placing the thorium atom at symmetry-inequivalent positions inside the cage. Geometry optimizations were performed using the PBE0 hybrid functional^23^ with Grimme’s D3 dispersion correction,^24^ the def2-SVP basis set for carbon,^25^ and a 60-valence-electron relativistic pseudopotential^26^ (MDF60) for thorium (PBE0-D3/def2-SVP/MDF60). The selected structure were also optimized using the PBE-D3 and B3LYP-D3 functionals^27,28^ to assess the functional dependence.

The lowest-energy conformers of each cage isomer were reoptimized using a larger def2-TZVP basis set for carbon,^25^ retaining the MDF60 pseudopotential (PBE0-D3/def2-TZVP/MDF60). Harmonic frequency calculations confirmed that all reported structures correspond to true minima.

### 2.2 Bonding Analysis

Chemical bonding was analyzed within the Quantum Theory of Atoms in Molecules (QTAIM)^9^ framework using AIMAll.^29^ Localization indices (LI) were used to estimate the thorium oxidation state, and delocalization indices (DI) were used to quantify Th–cage bonding. The LIs and DIs are obtained by integrating the electron density over the corresponding basins and correspond to the number of electrons localized on an atom (LI) and electron pairs shared between two atoms (DI). The oxidation state of Th was then estimated from the difference between the atomic number of Th and the calculated LI on Th. Analyses were performed on geometries optimized at the PBE0-D3/def2-TZVP/MDF60 level.

### 2.3 Calculations of UV–Vis–NIR Spectra

Electronic excitation energies and oscillator strengths for first 50 excited states were calculated using time-dependent density functional theory (TD-DFT) for the experimentally observed Th@*C*_2_(8)–C_84_ and Th@*C_s_* (15)–C_84_ isomers. Calculations employed the B3LYP^28^ and PBE0 ^23^ functionals, the 6-311++G** basis set^1^ for carbon and the MDF60 pseudopotential for thorium.^26^ The two lowest-energy transitions were additionally evaluated using the BHandHLYP,^30^ M06-2X,^31^ LC-BLYP,^32^ and CAM-B3LYP^33^ functionals. Solvent effects in CS_2_ were modeled using the SMD^34^ and CPCM polarized continuum models. Simulated spectra were generated by Gaussian convolution (HWHM = 0.1 eV).

### 2.4 Calculations of NMR Parameters

^13^C NMR shielding constants were calculated using Gaussian 16 ^22^ and ADF (AMS 2023.101).^35^ Scalar-relativistic calculations were performed at the PBE0/IGLO-III^36^ level with the MWB60 pseudopotential for thorium. Relativistic effects were assessed using scalar ZORA and spin–orbit ZORA at the PBE0/TZ2P level using CPCM implicit solvation, as implemented in ADF.^37,38^

The ^13^C chemical shifts were obtained from calculated isotropic shielding constants σ_iso_ according to formula

$$\delta =(\sigma_{\mathrm{ref}}-\sigma )/(1-\sigma_{\mathrm{ref}}\times 10^{-6}-)+\delta_{\mathrm{C60‐TMS}}$$

where C_60_ was used as a secondary reference system calculated at the same levels as Th@C_84_ and δ_C60‑TMS_ = 143.15 ppm is the experimental chemical shift of C_60_ vs TMS. Spectra were generated from the calculated ^13^C chemical shifts using MestReNova, Version 15.1.0.

## 3 Results

### 3.1 Relative Energy Ordering of Th@C_84_ Isomers

To determine the true energetic ordering of the Th@C_84_ isomers, we systematically explored the conformational space associated with different positions of the thorium atom inside all 24 isolated pentagon rule (IPR) C_84_ cages. For each cage isomer, four to seven symmetry-inequivalent starting geometries were generated by placing the thorium atom at distinct positions within the cage. Geometry optimization yielded multiple local minima for most cage isomers.

At the PBE0-D3/def2-SVP/MDF60 level, the relative energies of conformers belonging to a single cage isomer span a wide range, often exceeding 200 kJ·mol^–1^. Importantly, optimizations starting from geometries more than approximately 100 kJ·mol^–1^ above the lowest-energy conformer did not relax to the global minimum. Instead, they converged to higher-energy local minima, indicating the presence of deep and well-separated basins on the intramolecular potential energy surface.

Among the 24 IPR Th@C_84_ isomers considered in our previous study, only six, namely isomers 4, 10, 11, 12, 13, and 20 were confirmed to correspond to true global minima of their respective cages. The remaining isomers correspond to higher-energy conformers that are not directly connected to the global minimum via downhill relaxation pathways. This behavior reflects the strong directional covalent interaction between thorium and the carbon cage, which effectively localizes the metal atom and inhibits structural reorganization during geometry optimization.

To assess the dependence of the located minima on the choice of exchange–correlation functional, all starting geometries were additionally optimized using the PBE-D3 and B3LYP-D3 functionals at the same basis-set level. In all cases, the same sets of local minima were recovered, with only minor variations in the relative energies and geometries. No additional geometrically different low-energy conformers were identified. Harmonic frequency calculations confirmed that all reported structures correspond to true minima.

The lowest-energy conformer of each cage isomer was subsequently reoptimized using a larger def2-TZVP basis set (PBE0-D3/def2-TZVP/MDF60). The relative electronic energies obtained at this level are shown in Figure 1, along with the corresponding values from our previous study. The revised energy ordering clearly identifies Th@*C*_2_(8)–C_84_ and Th@*C*_*s*_(15)–C_84_ as the lowest-energy IPR isomers, in agreement with experimental observations.^3^

**Figure 1:**
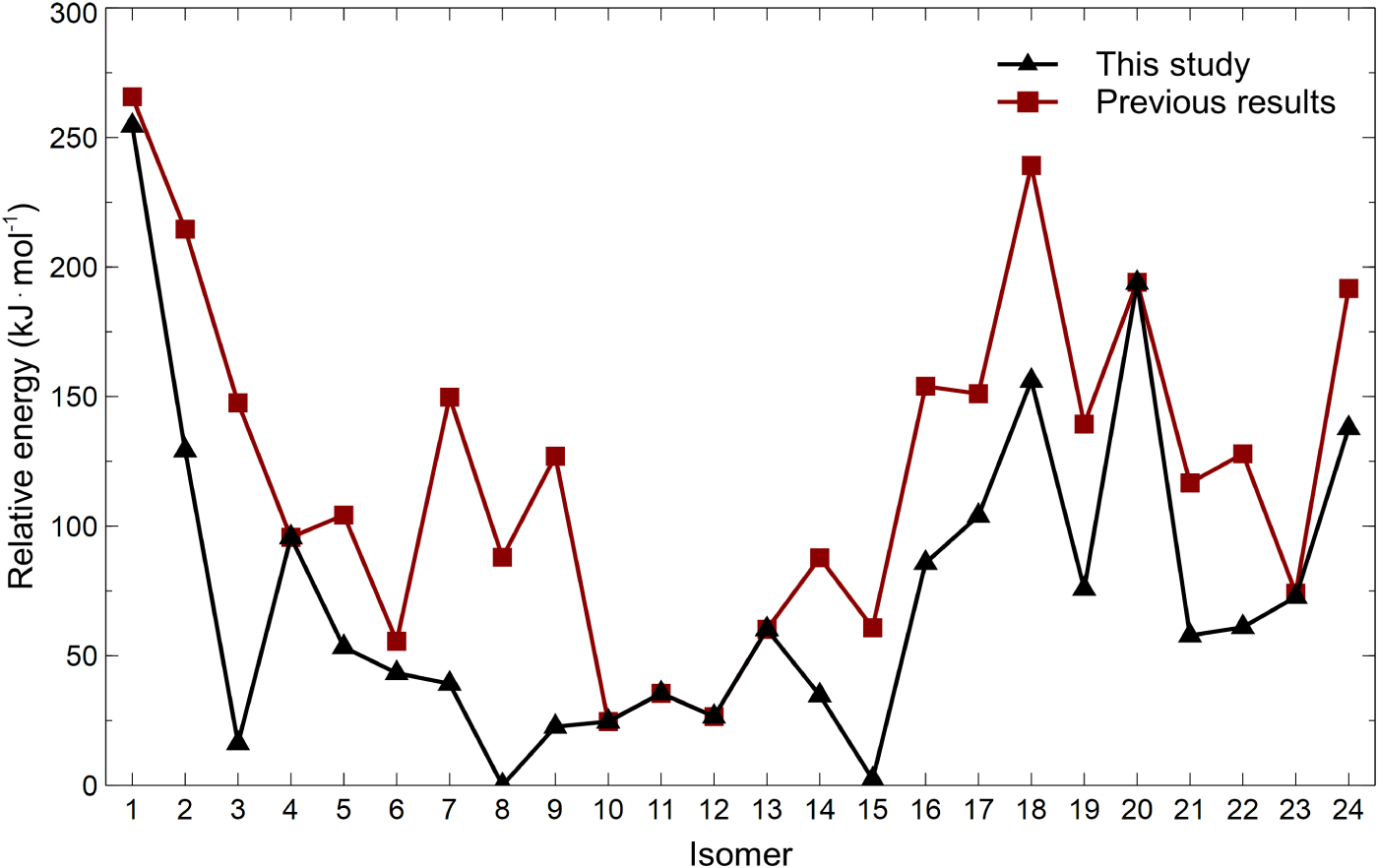
Relative electronic energies (kJ·mol^–1^) of the lowest-energy conformers of 24 IPR Th@C_84_ isomers. The energies from the present study (PBE0-D3/def2-TZVP/MDF60) are compared with those reported previously (PBE0-D3/def2-TZVP/MWB60).

The distributions of the energies of all the located conformers for each IPR isomer are summarized in Figure 2. The large energetic separation between distinct conformers highlights the importance of extensive conformational sampling in Th@C_84_. The failure of some optimizations to reach the global minimum in earlier studies is thus attributed to convergence into higher-energy conformers rather than to deficiencies in the electronic structure methods employed.

**Figure 2:**
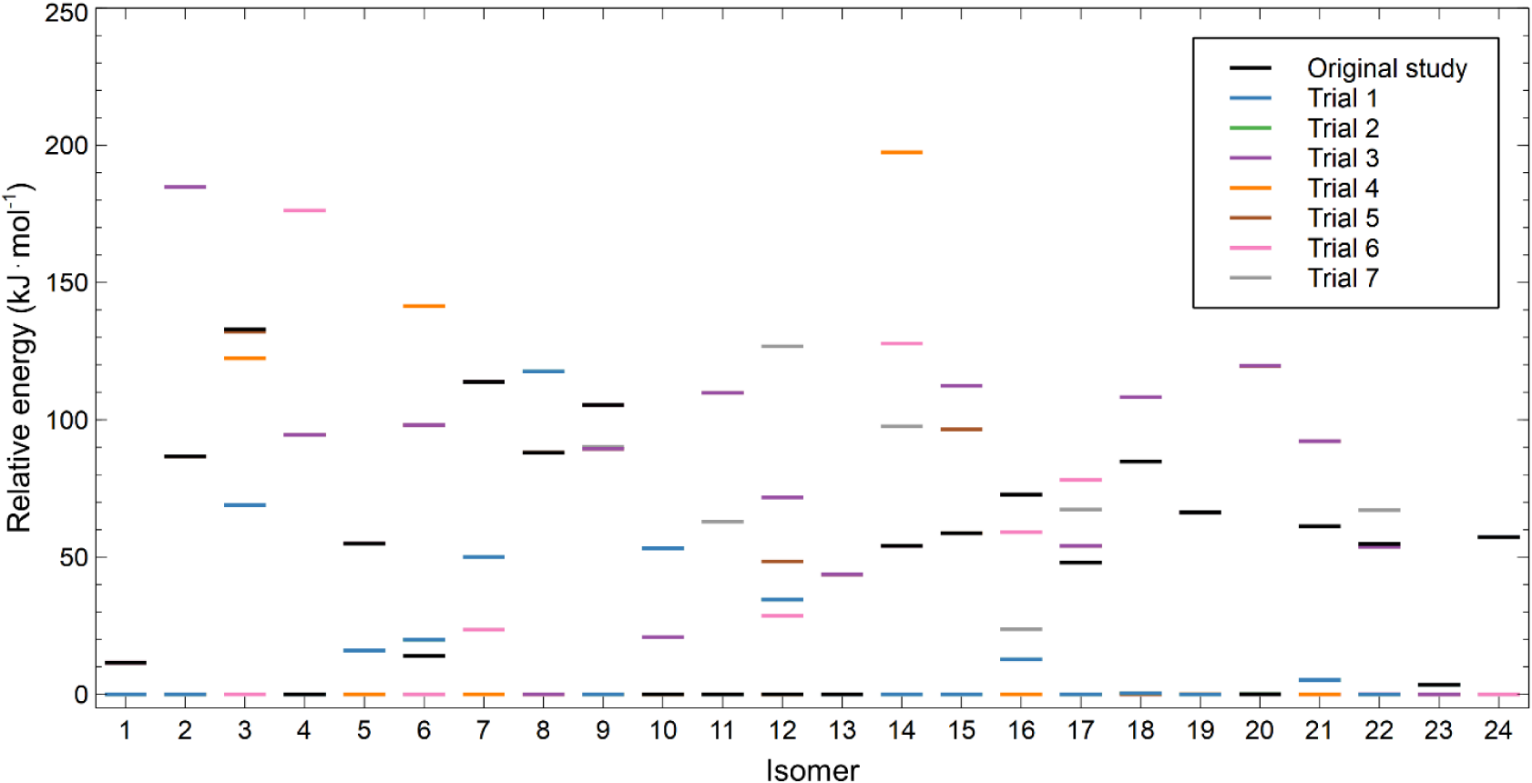
Relative electronic energies (kJ·mol^–1^) of all located conformers of the 24 IPR Th@C_84_ isomers calculated at the PBE0-D3/def2-SVP/MDF60 level. For each cage isomer, the energy of the lowest-energy conformer is set to zero. Each trial differs by the starting geometry. Note that some of the starting geometries converge to the same minima.

### 3.2 Chemical Bonding Analysis

Chemical bonding was analyzed for all located conformers of the experimentally observed Th@*C*_2_(8)–C_84_ and Th@*C*_*s*_(15)–C_84_ isomers using the Quantum Theory of Atoms in Molecules (QTAIM). The results are summarized in Table 1.

**Table 1:** The Relative Energies of the Given Conformers, in kJ·mol^–1^, Oxidation States of the Thorium Atom, OS, Calculated as the Difference between the Localization Index, LI, and Atomic Number of Thorium^a^

	Th@C_2_(8)–C_84_					**Th@C**_***s***_ **(15)–C_84_**				
Conformer	A	B	C	D	original	A	B	C	D	original
**Relative energy**	117.6	0.0	0.0	88.0	88.0	0.0	112.4	58.7	96.5	58.7
**OS**	4.2	4.2	4.2	4.2	4.2	4.2	4.2	4.2	4.2	4.2
**DI (Th–C)**	4.0	3.8	3.8	3.8	3.8	3.8	4.0	4.0	3.9	3.9
**Bonding pattern**	HHP	H	H	H	HH	H	HHP	HH	HH	HH
r (Th–C)	2.37	2.44	2.44	2.39	2.39	2.44	2.37	2.38	2.36	2.38

a The sum of the delocalization indices, DI (Th–C), between the thorium atom and all the carbon atoms of the cage, bonding patterns between thorium atom and carbon atoms, and the shortest Th–C distances, in Å, at the PBE0/def2-SVP/MDF60 level. H denotes bonding pattern with thorium atom in the middle of a carbon hexagon, HH in between two hexagons, HHP in between two hexagons and one pentagon.

Across all conformers, the calculated oxidation state of thorium, obtained from the difference between its atomic number and the localization index (LI), is close to **+4**, in agreement with previous experimental and theoretical studies. The sum of delocalization indices (DI) between thorium and the carbon cage ranges from **3.8 to 4.0**, with the largest individual Th–C contributions between **0.3 and 0.4**, indicating pronounced covalent metal–cage interactions.

Importantly, only minor variations in the oxidation state, total Th–C delocalization, and shortest Th–C distances are observed between different conformers of a given cage isomer. The shortest Th–C distances fall within a narrow range of **2.36–2.44 Å** for all structures considered. These results indicate that the electronic structure of the Th–cage interaction remains essentially unchanged across conformers.

Despite their similar bonding characteristics, distinct geometric bonding motifs are observed, corresponding to thorium positioned near one hexagon (H), between two hexagons (HH), or between two hexagons and one pentagon (HHP). The lowest-energy conformers of both isomers favor coordination with a single hexagon. The representative bonding motifs for the lowest-energy conformers are shown in Figure 3.

**Figure 3:**
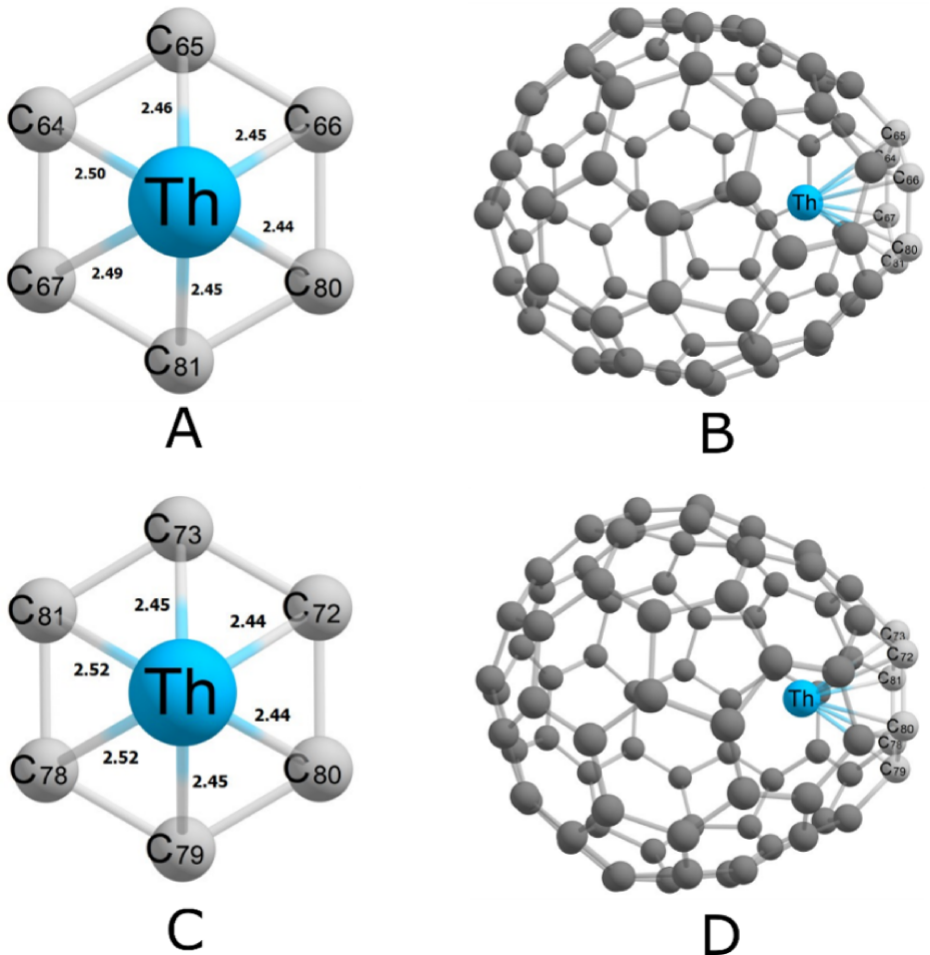
Representative structural motifs of the lowest-energy conformers of Th@*C*_2_(8)–C_84_ (A, B) and Th@*C_s_* (15)–C_84_ (C, D), illustrating the different thorium coordination environments within the carbon cage.

The absence of significant electronic differences between conformers indicates that the energetic separation between them arises primarily from geometric constraints imposed by strong, directional Th–cage covalent bonding rather than from changes in the underlying electronic structure. This finding supports the conclusion that conformational trapping in Th@C_84_ originates from the topology of the intramolecular potential energy surface, rather than from variations in metal–cage bonding. Similar behavior was observed for the Ti@C_70_ system in our previous study, where two different minimum-energy conformers were identified.^39^

### 3.3 Theoretical vs Experimental Absorption Spectra of Th@*C*_2_(8)–C_84_ and Th@*C*_*s*_(15)–C_84_

The calculated UV–vis–NIR absorption spectra of the experimentally observed Th@*C*_2_(8)–C_84_ and Th@*C*_*s*_(15)–C_84_ isomers were compared with the available experimental data to further validate the revised structural assignments. The calculated and experimental spectra are presented in Figure 4.

**Figure 4:**
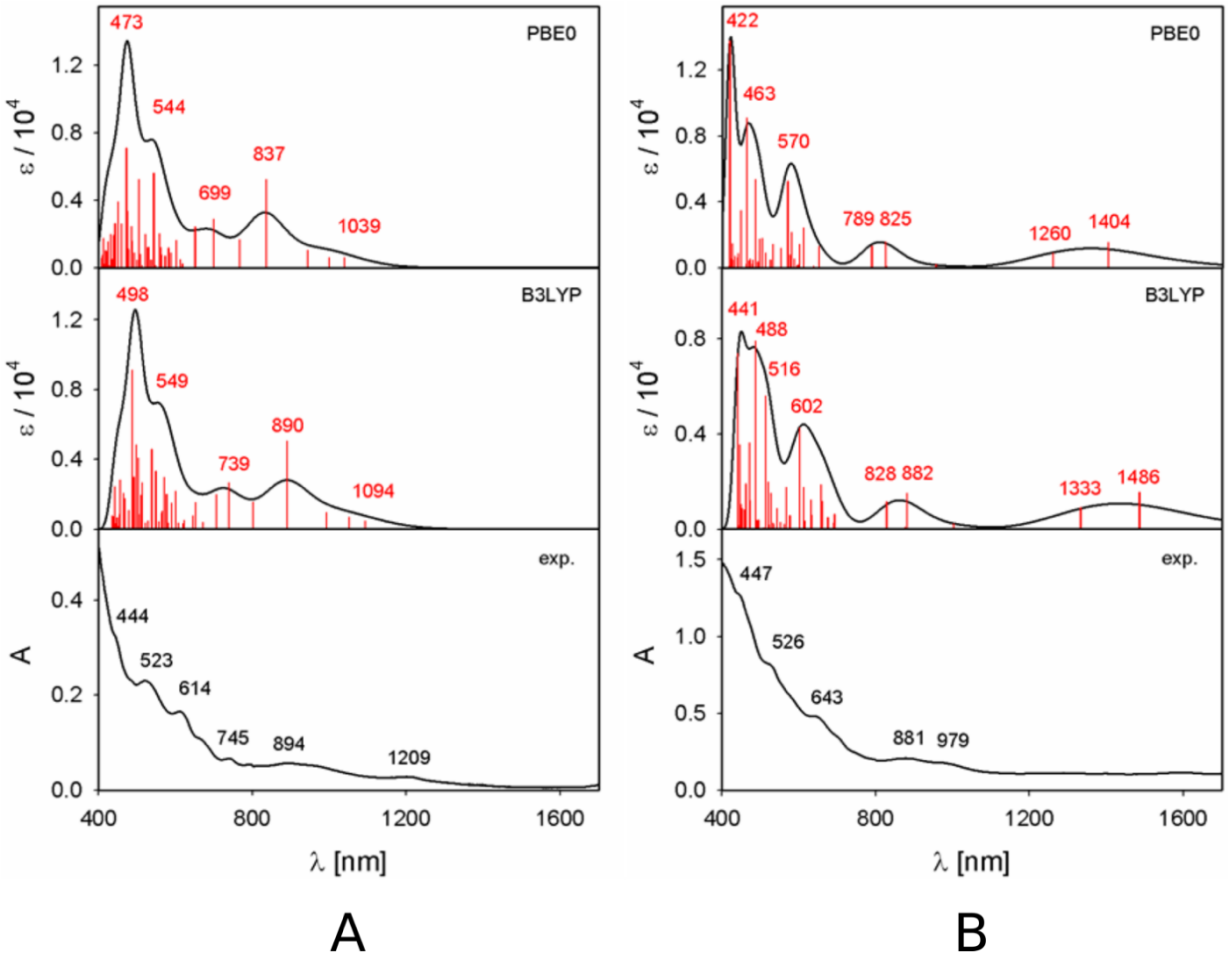
Comparison of experimental and calculated UV–vis–NIR absorption spectra of (A) Th@*C*_2_(8)–C_84_ and (B)­Th@*C_s_* (15)–C_84_.

Experimentally, Th@*C*_2_(8)–C_84_ exhibits six major absorption bands at 444, 523, 614, 745, 894, and 1209 nm, whereas Th@*C*_*s*_(15)–C_84_ displays five well-resolved bands at 447, 526, 643, 881, and 979 nm. TD-DFT calculations performed using the B3LYP and PBE0 functionals reproduce the overall spectral features in the visible region for both isomers, except at the low-energy end. In all cases, the calculated transitions are slightly blue-shifted relative to the experiment, with B3LYP generally yielding closer agreement than PBE0.

Larger deviations are observed for the low-energy transitions in the near-infrared region. For Th@*C*_2_(8)–C_84_, the lowest experimental band at 1209 nm is predicted at higher energy by both functionals. For Th@*C_s_* (15)–C_84_, no corresponding experimental band has been reported in this region; however, TD-DFT calculations predict low-energy transitions at longer wavelengths (1400–1500 nm) than those for Th@*C*_2_(8)–C_84_. Closer examination of the experimental spectrum, see the amplified region in Figure 5, reveals signs of a low-intensity band in this region, suggesting the possible presence of an as-yet undetected low-energy transition.

**Figure 5:**
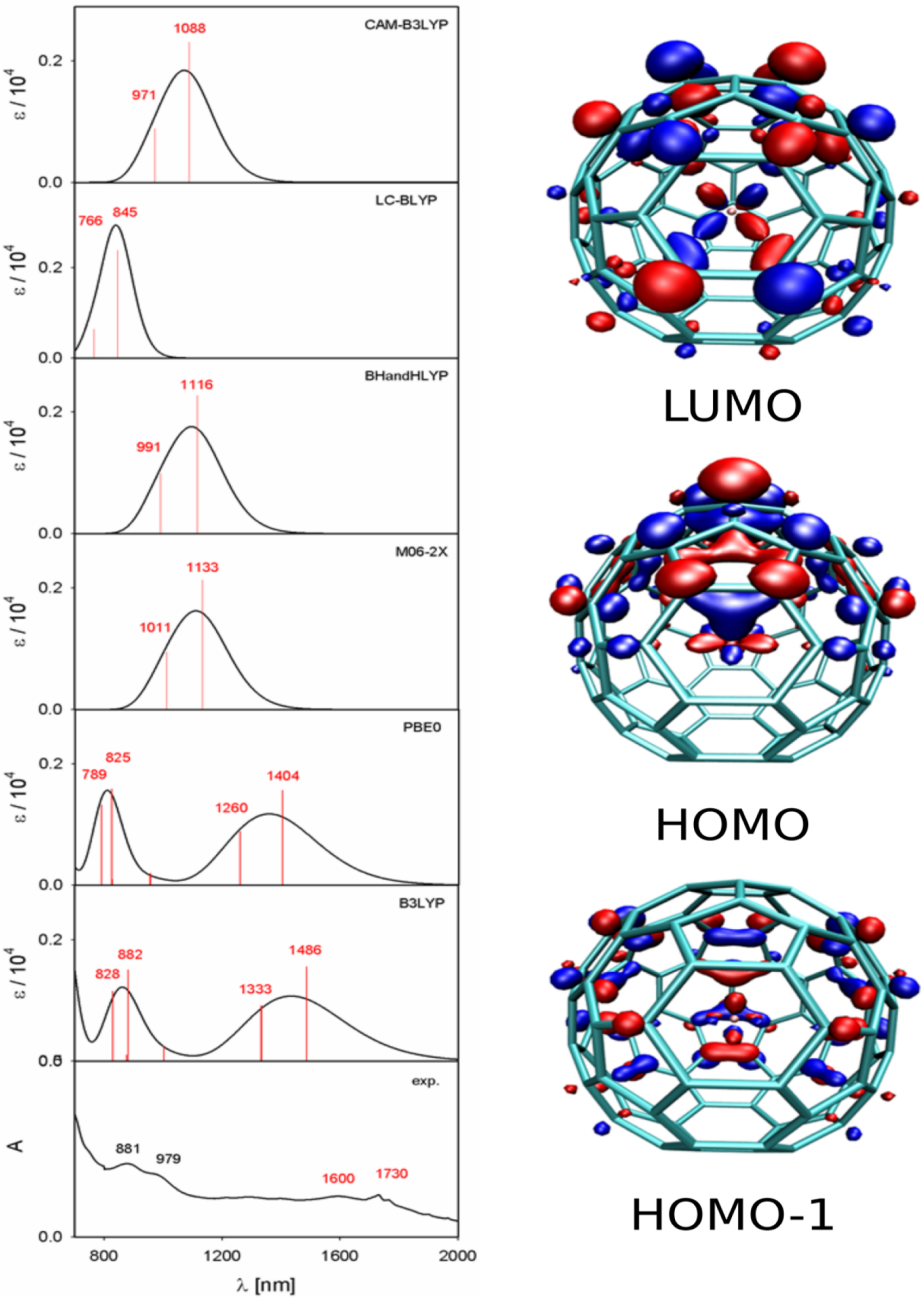
Selected lowest-energy electronic transitions of Th@*C_s_* (15)–C_84_. The experimental and calculated UV–vis–NIR spectra in the 700–2000 nm region are shown on the left, and isosurface representations of the HOMO–1, HOMO, and LUMO, calculated at the B3LYP/6-311++G**/ECP level (isovalue = 0.04 e Å^–3^), are shown on the right.

To further assess the robustness of the predicted low-energy excitations, the two lowest-energy transitions were recalculated using the BHandHLYP, M06-2X, LC-BLYP, and CAM-B3LYP functionals. All the tested functionals in Figure 5 predict these transitions at higher energies than those observed experimentally, indicating a systematic limitation of single-reference TD-DFT for describing low-lying excitations in these systems. Inclusion of solvent effects (CS_2_) using the SMD and CPCM continuum models has only a minor influence on higher-energy transitions, but strongly shifts the lowest-energy excitations to longer wavelengths (Figure 6).

**Figure 6:**
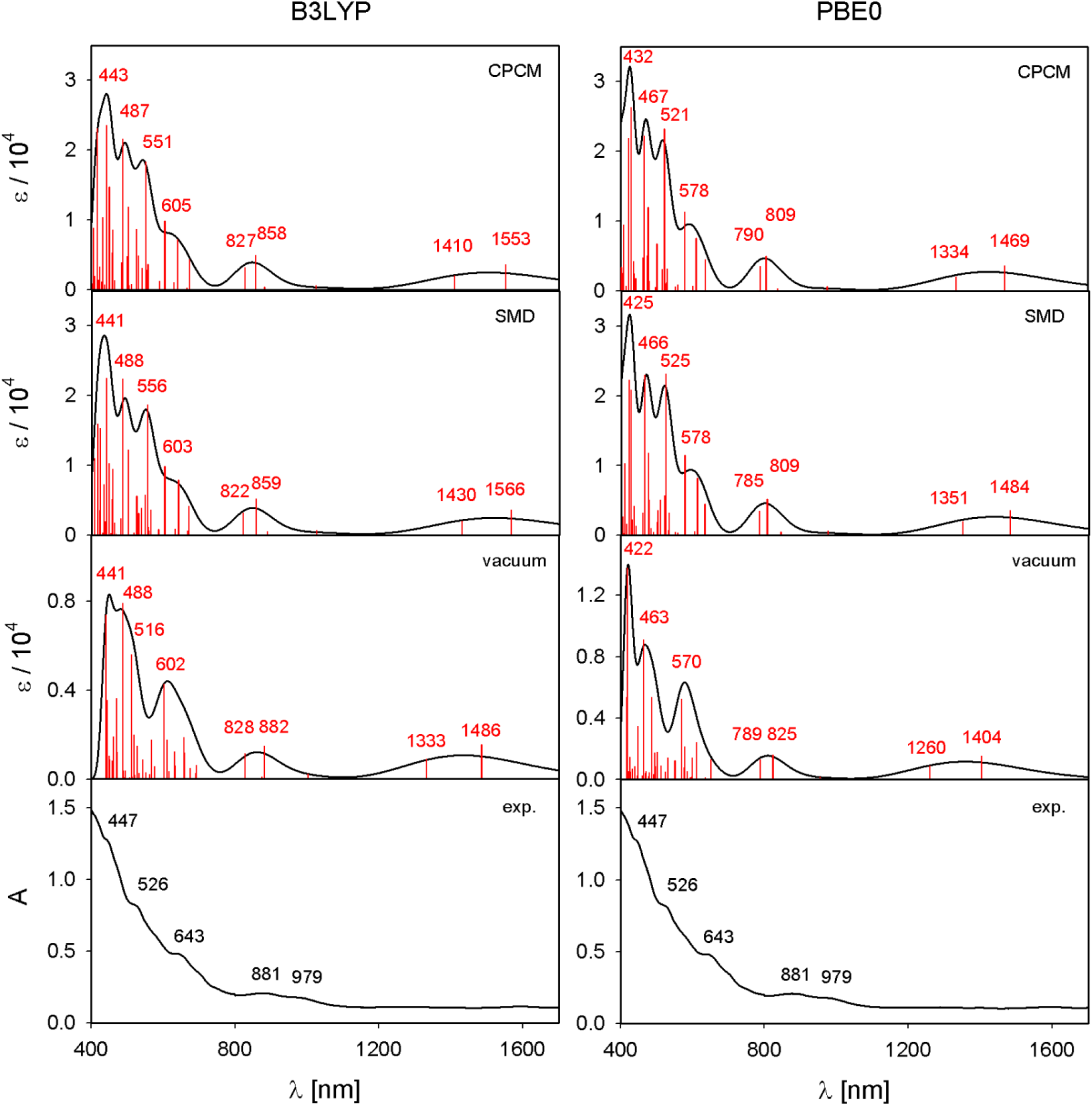
Comparison of experimental UV–vis absorption spectra of Th@*C_s_* (15)–C_84_ and theoretical spectra calculated at the B3LYP/6-311++G**/ECP (left) and PBE0/6-311++G**/ECP (right) levels in vacuum and using two continuous solvent models (SMD, CPCM).

Overall, the calculated UV–vis spectra show good agreement with experiment over most of the spectral range and correctly reproduce the qualitative differences between the two Th@C_84_ isomers. The main discrepancies observed for transitions in the near-infrared region are attributed primarily to methodological limitations in describing low-energy excitations at the TD-DFT level, rather than to deficiencies in the underlying structural models. Note that the difference between experimental and theoretical electronic excitation energies (cf Table S1 in SI) is relatively constant on an absolute energy scale (∼0.1 eV), whereas this is not the case on the relative wavelength scale. Consequently, for low-energy excitations the same absolute deviation represents a much larger fraction of the excitation energy, leading to disproportionately large wavelength errors in the NIR region (i.e., units to tens of nanometers for UV–vis transitions, tens to hundreds of nanometers for NIR transitions). Other factors, such as potential multireference character of the system, or vibronic splitting of the studied NIR transitions are likely minor.

Table S1 lists the dominant orbital contributions for each experimentally observed band. The lowest-energy transition corresponds primarily to the HOMO → LUMO excitation. Representations of these orbitals for Th@*C_s_* (15)–C_84_ are shown in Figure 5. Similarly, the second transition in Th@*C*_2_(8)–C_84_ and Th@*C_s_* (15)–C_84_ corresponds primarily to HOMO – 1 → LUMO + 1 excitation. A more detailed orbital analysis reveals that the lowest transitions are dominated by π–π^∗^ transitions of the fullerene cage with minor participation of the 6d/7s/5f orbitals of thorium, see Figure 5. The contribution of thorium to the lowest transitions can be demonstrated by a simple numerical experiment in which the thorium inside the cage is moved away from or toward the optimized position, and the change in the excitation energy of the first transition is monitored. Table S2 shows the dependence of the excitation energy of Th@*C_s_* (15)–C_84_ on the Th-cage distance. For example, upon moving from an empty cage to a cage with artificially short Th-cage distance, a blue shift of the HOMO → LUMO excitation is observed in Table S2.

### 3.4 Predicted NMR Spectra

To further support the revised structural assignments, we calculated the ^13^C NMR chemical shifts for the experimentally observed Th@*C*_2_(8)–C_84_ and Th@*C_s_* (15)–C_84_ isomers, respectively. The predicted ^13^C NMR spectra of the two isomers in Figure 7 exhibit distinct signal distributions, reflecting their different cage symmetries and thorium positions. For Th@*C*_2_(8)–C_84_, the calculated signals are more evenly distributed over the chemical shift range, whereas Th@*C_s_* (15)–C_84_ displays a more clustered pattern of resonances. These differences suggest that the two isomers can be readily distinguished by ^13^C NMR spectroscopy.

**Figure 7:**
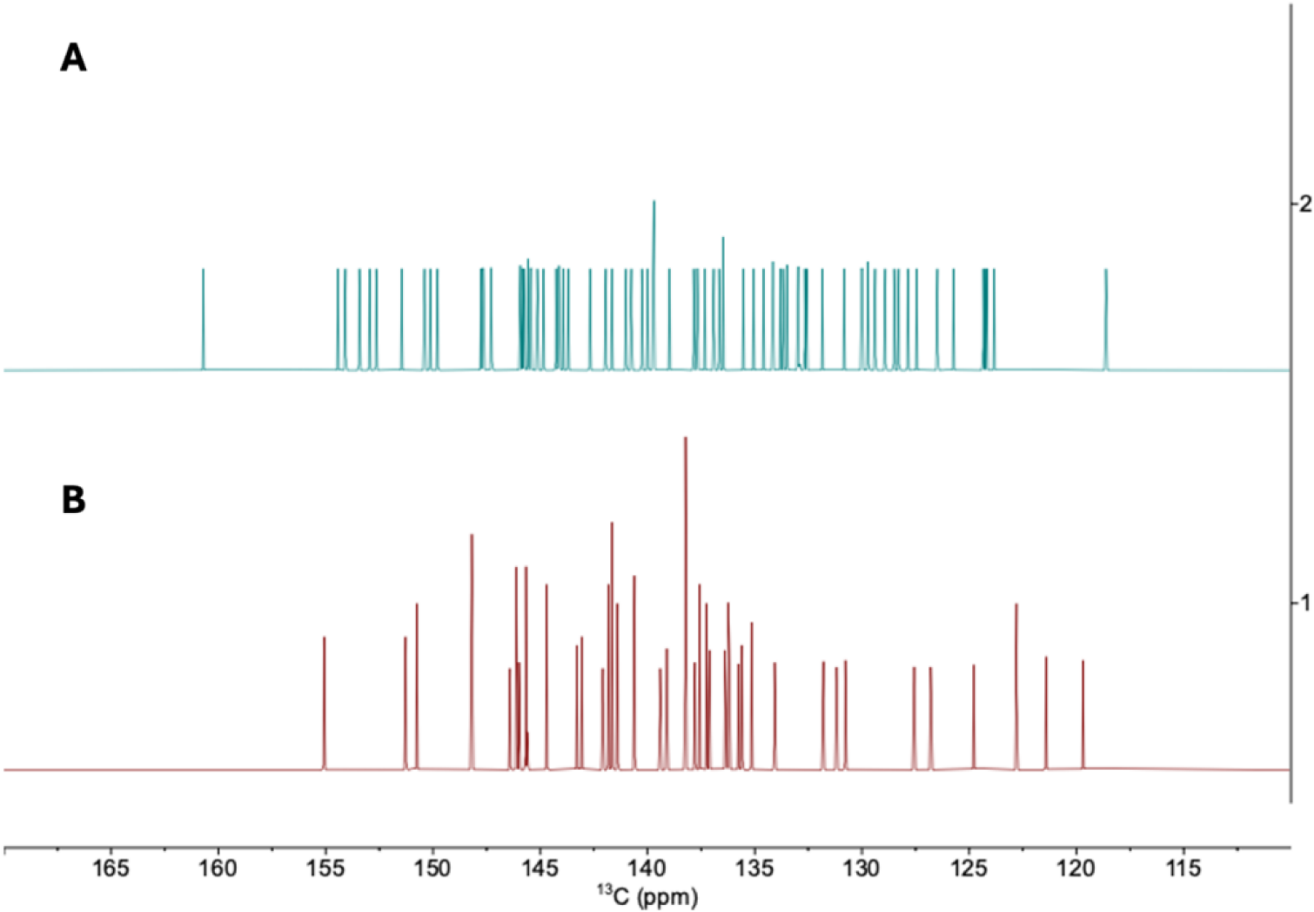
Calculated ^13^C NMR spectra of (A) Th@*C*_2_(8)–C_84_ and (B) Th@*C_s_* (15)–C_84_.

The relativistic effects on the calculated ^13^C chemical shifts were assessed using the scalar ZORA and spin–orbit ZORA approaches. As summarized in Figure 8, the spin–orbit contributions are generally small and exceed 1 ppm only for a limited number of carbon atoms closest to the thorium center. The differences between the calculations employing relativistic pseudopotentials and ZORA-based treatments are likewise minor and rarely exceed 1 ppm.

**Figure 8:**
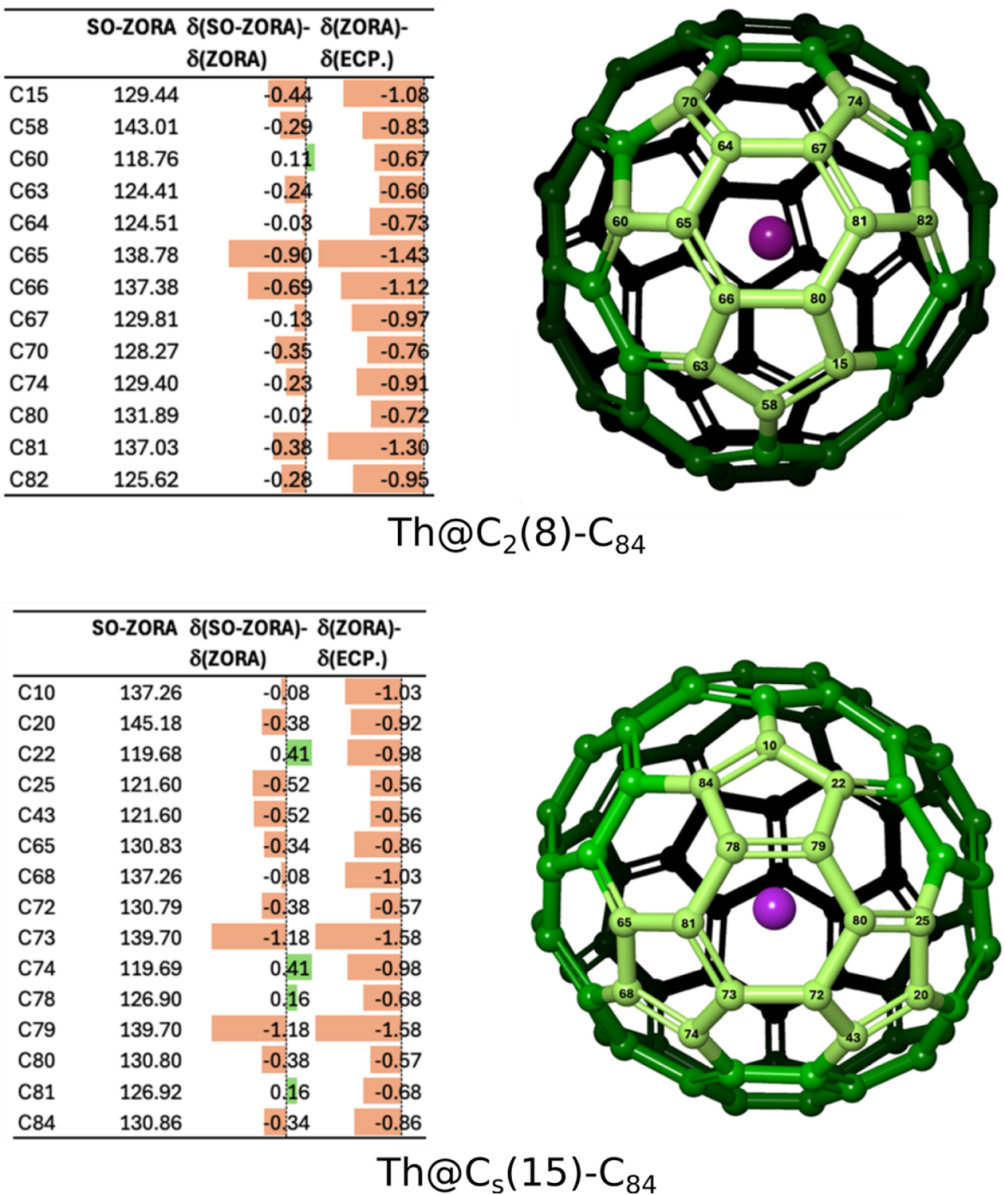
Predicted ^13^C chemical shifts of Th@*C*_2_(8)–C_84_ and Th@*C_s_* (15)–C_84_ at scalar relativistic (ECP and ZORA) and spin–orbit relativistic (SO-ZORA) levels. Deviations arising from different relativistic approximations are indicated. Only carbon atoms within 3 Å of the thorium atom are shown, corresponding atom labels are highlighted in the structural representations.

Overall, the calculated ^13^C NMR signatures are governed primarily by the molecular geometry and cage symmetry, while the relativistic effects associated with the thorium atom play a secondary role. These results further corroborate the structural assignments of Th@*C*_2_(8)–C_84_ and Th@*C_s_* (15)–C_84_ and support the reliability of optimized geometries.

## 4 Conclusions

We reevaluated the energetic ordering of Th@C_84_ endohedral fullerene isomers in light of recent experimental findings. Systematic exploration of the conformational space associated with different thorium positions inside the carbon cage reveals that the previously proposed Th@*C_s_* (10)–C_84_ structure does not correspond to the true global minimum. Instead, the experimentally observed Th@*C*_2_(8)–C_84_ and Th@*C_s_* (15)–C_84_ isomers are confirmed as the lowest-energy structures when proper conformational sampling is employed.

The earlier misassignment is traced to convergence into higher-energy local minima arising from strong, directional Th–cage covalent interactions, which partition the intramolecular potential energy surface into multiple deep basins. This behavior establishes conformational isomerism as an intrinsic and chemically relevant feature of the actinide endohedral fullerenes.

Bonding analysis confirms a robust Th­(IV) oxidation state with pronounced metal–cage covalency across the conformers. The reevaluated UV–vis–NIR spectra for the experimentally observed isomers are in good agreement with the available measurements. The predicted ^13^C NMR spectra distinguish the two experimentally observed isomers and show only minor spin–orbit relativistic effects on the calculated ^13^C chemical shifts. More generally, our results demonstrate that extensive conformational sampling is essential for the reliable theoretical characterization of actinide metallofullerenes and related systems with strong metal–cage covalency.

## Supplementary Material



## Data Availability

All calculation details and data supporting the findings of this study can be found within the paper, or at the OSF repository (doi: 10.17605/OSF.IO/8FD9U; https://osf.io/8fd9u/overview).

## Notes

Table S1 lists the observed and calculated electronic transitions in Th@*C*_*s*_(15)–C_84_ and Th@*C*_2_(8)–C_84_ systems; Table S2 contains the dependence of excitation spectrum of Th@*C_s_* (15)–C_84_ on Th-cage distance (PDF)
