# Supplementary material for: Th@C84 Revisited: Lost in the Corners of One’s Own Cage
Source: J Phys Chem A. 2026 May 4;130(19):3793–801. doi: 10.1021/acs.jpca.6c00909 (PMC13181788; doi:10.1021/acs.jpca.6c00909)
Supplement: Supplementary file 1 [file jp6c00909_si_001.pdf]

Supporting Information for

$Th@C_{84}$ revisited: Lost in the corners of one's own cage

Jakub Kaminský,[1,2,] Adam Jaroš,[1] Michal Straka[1,]**

[1]Institute of Organic Chemistry and Biochemistry of the Czech Academy of Sciences, Flemingovo nám. 2, CZ–16610 Prague, Czech Republic.
[2]Department of Chemistry of Natural Compounds, University of Chemistry and Technology Prague, Technická 5, 166 28 Prague, Czech Republic.

Table S1: Comparison of experimentally observed and calculated[a] electronic transitions in Th@C_s(15)–C_{84} and Th@C_2(8)–C_{84}

**Th@C_s(15)–C_{84}**	**$E_{exp.}$**		**$E_{calc.}$ (gas)**		**f**	**$E_{calc.}$ (CS_2)**		**f**	**Dominating transitions**
	nm	eV	nm	eV		nm	eV		
1	447	2.77	441	2.81	0.028	443	2.80	0.085	HOMO − 9→LUMO
2	526	2.36	514	2.41	0.021	551	2.25	0.066	HOMO→LUMO + 3
3	643	1.93	602	2.06	0.016	605	2.05	0.036	HOMO − 6→LUMO
4	881	1.41	828	1.50	0.004	827	1.50	0.012	HOMO − 3→LUMO
5	979	1.27	882	1.41	0.006	858	1.44	0.018	HOMO→LUMO + 1
6	1600	0.77	1333	0.93	0.003	1410	0.88	0.007	HOMO − 1→LUMO
7	1730	0.72	1486	0.83	0.006	1553	0.80	0.013	HOMO→LUMO

**Th@C_2(8)–C_{84}**	**$E_{exp.}$**		**$E_{calc.}$ (gas)**		**f**	**$E_{calc.}$ (CS_2)**		**f**	**Dominating transitions**
	nm	eV	nm	eV		nm	eV		
1	444	2.79	498	2.49	0.018	438	2.83	0.024	HOMO − 3→LUMO + 2
2	523	2.37	549	2.26	0.012	529	2.34	0.016	HOMO − 5→LUMO + 1
3	614	2.02	601	2.06	0.008	613	2.02	0.019	HOMO − 3→LUMO
4	745	1.66	739	1.68	0.010	724	1.71	0.029	HOMO − 2→LUMO + 1
5	894	1.39	890	1.39	0.019	860	1.44	0.041	HOMO − 1→LUMO + 1
6	1209	1.03	1094	1.13	0.002	1118	1.11	0.012	HOMO→LUMO

[a]The theoretical excitation energies (*E* in nm or eV) oscillator strengths (*f*) and dominant orbital transitions for the selected transitions are also listed. Calculations were performed at the B3LYP/6-311++G**(MDF60) level in the gas phase or with the CPCM (CS_2) model. The red-highlighted transitions have not yet been reported.

Table S2. The dependence of the theoretical* excitation energy of Th@C_s(15)–C_{84} on the C72-Th distance.

**r(C72-Th)**	**$E_{calc.}$**
2.0	1298
2.44	1553
3.0	2163
no Th	3375

*Calculations were performed at the B3LYP/6-311++G**(MDF60)/CPCM level.
